# Nobiletin reduces 5-FU-induced lung injury with antioxidative, anti-inflammatory and anti-apoptotic activities

**DOI:** 10.1007/s00210-024-03773-6

**Published:** 2025-01-17

**Authors:** Gözde Atila Uslu, Hamit Uslu, Taha Abdulkadir Çoban, Mustafa Özkaraca, Ali Sefa Mendil, Serpil Aygörmez

**Affiliations:** 1 Department of Physiology, Faculty of Medicine, Erzincan Binali Yıldırım University, Erzincan, Turkey; 2 Department of Biochemistry, Faculty of Medicine, Erzincan Binali Yıldırım University, Erzincan, Turkey; 3 Department of Pathology, Faculty of Veterinary Medicine, Cumhuriyet University, Sivas, Turkey; 4 Department of Pathology, Faculty of Veterinary Medicine, Erciyes University, Kayseri, Turkey; 5 Department of Biochemistry, Faculty of Veterinary Medicine, Kafkas University, Kars, Turkey

**Keywords:** 5-Fluorouracil, Nobiletin, Inflammation, Apoptosis, Oxidative stress, Lung damage

## Abstract

Like other chemotherapeutic agents, 5-fluorouracil (5-FU) targets cancerous cells, but it also causes many unwanted side effects on healthy tissues and cells. Based on the undesirable effects of 5-FU, the aim of this study was to determine how 5-FU affects lung tissue and whether nobiletin has any protective effect. The study consisted of negative control, Nobiletin, 5-FU and Nobiletin + 5-FU groups. Nobiletin and Nobiletin + 5-FU groups received 10 mg/kg Nobiletin i.g. for 7 days. On day 8, 100 mg/kg 5-FU was administered i.p. to 5-FU and Nobiletin + 5-FU groups. Biochemical and immunohistochemical analyses were performed on the lung tissues dissected at the end of the study. 5-FU caused growth retardation, disturbed the oxidant-antioxidant balance by increasing MDA levels and decreasing GSH levels, triggered cellular apoptosis by increasing Bax and caspase-3 levels and decreasing Bcl-2, also increased lung tissue inflammation and damage by increasing NFκB and IL-1β levels. However, it was determined that Nobiletin prevented the disruption of the oxidant-antioxidant balance, showed significant anti-apoptotic effects, especially by reducing Bax levels and partially modulating caspase-3 and Bcl-2 levels, and also exhibited anti-inflammatory effects by reducing NFκB and IL-1β levels and supported the normal development of animals. Our results showed that nobiletin pretreatment showed anti-inflammatory activity by inhibiting the NFκB pathway in 5-FU-induced lung injury, suppressed oxidative stress with its antioxidant activity and was effective in modulating cellular apoptosis with its anti-apoptotic activity. In conclusion, Nobiletin has been shown to have an important potential in reducing fluorouracil-induced tissue damage by acting through multiple pathways.

**Graphical Abstract:**

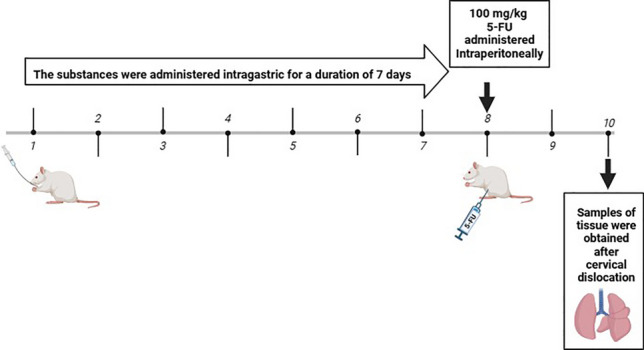

## Introduction

As in the past, one of the most widely used and basic methods of cancer treatment today is the use of chemotherapeutic agents (Safarpour et al. 2022a). However, it is necessary to be extremely careful in this use. As a matter of fact, chemotherapy treatment often causes very serious side effects. In addition to common side effects such as fever, nausea and vomiting, nephrotoxicity, neurotoxicity, hepatotoxicity and cardiotoxicity are among the most severe toxicities (Schirrmache 2019; Van Den Boogaard et al. 2022). Moreover, after cancer treatments, conditions such as depletion of the immune system and the development of resistance to anticancer drugs may be encountered, the dose of medication administered may be increased or the treatment protocol may be changed. Such situations can negatively affect patients who are already going through a troubled process (Sharma et al. 2024; Ward et al. 2020). 5-FU, a pyrimidine uracil analogue, has been used for many years, either alone or in combination with other chemotherapeutic agents, to treat a variety of malignancies, including colon, breast and skin cancer (Miura et al. 2010). The effect of 5-FU is mediated by its active metabolites fluoro-deoxyuridine monophosphate (FdUMP), fluorodeoxyuridine triphosphate (FdUTP) and fluorouridine triphosphate (FUTP). Studies have shown that these metabolites affect both DNA and RNA, disrupting the synthesis of genetic material and triggering apoptosis in cells (Sethy and Kundu 2021; Gelen et al. 2017). Nevertheless, the side effects of 5-FU on the lungs are also undeniable. 5-FU has been reported to cause severe pulmonary oedema, haemorrhage, thickening of the alveolar septum and reduction of alveoli (da Silva et al. 2023). It has also been shown to cause interstitial fibrosis, emphysema, mononuclear cell infiltration, congestion, increase in inter-alveolar tissue due to cellular infiltration, peribronchial lymphoid tissue hyperplasia, perivasculitis and epithelial cell shedding in bronchioles (Gedikli and Erbas 2021; Al-Hamdany and Al-Hubaity 2014a, b). It was also found that 5-FU increased nitric oxide synthase (NOS) and myeloperoxidase (MPO) while decreasing antioxidants such as catalase (CAT), superoxide dismutase (SOD) and glutathione peroxidase (GPX); in addition, it also has been affected the inflammation pathway by triggering excessive nuclear factor kappa-B (NFκB) production (Yu et al. 2022; Al-Asmari et al. 2016).

Chemotherapeutic drugs’ toxicity studies underline that these compounds create oxidative stress by producing excessive reactive oxygen species, severely promote inflammatory pathways and destroy healthy tissues and cells via apoptotic processes. For this reason, there has been a renewed interest in the search for antioxidant and anti-inflammatory compounds with the capacity to act on healthy and sick cells via multiple modes of action in order to protect against chemotherapy-induced tissue damage. Zamanian et al. (2024) emphasised that curcumin (one of the natural active ingredients) shows its protective effects against diabetic cardiomyopathy, lung damage and diabetic gastroparesis by inhibiting the increase in pro-inflammatory cytokine levels such as tumour necrosis factor-α (TNF-α), interleukin-1 beta (IL-1β) and interleukin-6 (IL-6) by regulating the NFκB signalling pathway and that regulatory effects on the NFκB pathway may be a therapeutic target in alleviating diabetic complications. Another study reported that nano-curcumin treatment decreased fasting plasma glucose, increased insulin sensitivity and decreased homeostatic model assessment of insulin resistance levels in patients with metabolic syndrome (Kheiripour et al. 2021). On the other hand, it is important to develop new therapeutic strategies that focus on preventing the inflammation and tumour-promoting effects caused by pathological NFκB activation, but it should be kept in mind that there are many factors that enable its activation and therefore complete inhibition cannot be achieved (Zhang et al. 2021). In a 2023 review on breast cancer, it was stated that ellagic acid showed anticancer activity by regulating various molecular mechanisms that play an active role in processes such as apoptosis, cell cycle, angiogenesis and metastasis (Golmohammadi et al. 2023). It has been reported that apigenin, a natural product belonging to the flavone class found in many plants, suppresses the cell cycle in the G2/M phase in colon cancer cells, inhibits apoptosis, autophagy formation and apigenin may be a promising agent for colon cancer treatment (Daneshvar et al. 2023).

Nobiletin, an important flavanoid that can be obtained from citrus peels, has become a popular active ingredient that has attracted the attention of researchers after it was found to have antioxidant, anti-inflammatory effects as well as cancer suppression and protection of normal cells against toxic agents (Moazamiyanfar et al. 2023; Yang et al. 2020; Luo et al. 2008; Nagappan et al. 2016). It has been reported that nobiletin is converted into demethylated metabolites such as 3′-demethylnobiletin, 4′ demethylnobiletin (4DN) and 3′,4′-didemethylnobiletin after oral ingestion and these metabolites have anti-inflammatory and anticancer effects by suppressing the expression of proinflammatory cytokines (Wu et al. 2015). In addition, it was emphasised that Nobiletin pretreatment increased GSH and SOD levels and decreased malondialdehyde (MDA) levels in hydrogen peroxide-induced cytotoxicity, and its antioxidant properties came to the fore by clearing reactive oxygen species (ROS) formation and had neuroprotective effects (Lu et al. 2010).

The purpose of this investigation was to ascertain if nobiletin might protect against 5-fluorouracil-induced lung damage based on effects that have been reported in the literature regarding its antioxidative, anti-inflammatory and anticancer properties.

## Material and methods

### Experimental design

The rats used in the study (28 2-month-old female Sprague–Dawley 181 ± 7 g) were obtained from Erzincan Binali Yıldırım University Experimental Animals Application and Research Centre. Animals were divided into 4 groups with 7 animals in each group. The initial weights of the animals were determined before starting the substance applications. Throughout the study, all animals were housed in cages with standard light–dark cycles and free access to food and water. The study groups were formed as follows.

- Negative control group: In order to ensure standardisation, 1% DMSO was administered intragastrically (i.g.) for 7 days. On the 8th day, intraperitoneally (i.p.) saline was administered for standardisation.
- Nobiletin group: Nobiletin (dissolved in 1% DMSO) at a dose of 10 mg/kg (Güvenç et al. 2020) was administered i.g. for 7 days. On the 8th day, i.p. saline was administered for standardisation.
- 5-FU group: In order to ensure standardisation, 1% DMSO was administered i.g. for 7 days. On the 8th day, 100 mg/kg (Safarpour et al. 2022a, b) single dose of 5-FU i.p. was administered.
- Nobiletin + 5-FU group: Nobiletin at a dose of 10 mg/kg was administered i.g. for 7 days. On the 8th day, 100 mg/kg single dose of 5-FU i.p. was administered.

### Sample collection and preparation for analyses

Rats whose final body weights were determined 48 h after a single dose of 5-FU administration were anaesthetised and euthanasia was performed by cervical dislocation. Lung tissues were then removed and weighed. Lung weights were normalised according to the percentage of body weight of the animals (relative organ weight = organ weight/animal final body weight × 100) (Ugwah-Oguejiofor et al. 2019). While the left lung was placed in 10% formaldehyde for histopathological examinations, the right lung was preserved in appropriate conditions for biochemical analyses.

### Chemicals and biochemical analyses

5-FU (1000 mg/20 mL injectable solution) was supplied from Koçak Pharmaceuticals (Istanbul, Turkey). Nobiletin (cas number: 478–01–3) was obtained from Toronto Research Chemicals Inc. (Canada). Additionally, trichloroacetic acid (cas number: 76–03–9 Supelco-Germany), Trizma (cas number: 77–86-1 Merck-USA), 2-nitrobenzoic acid (cas number: 69–78-3 Merck-Germany), l-glutathione reduced (cas number: 70–18-8 Merck-China), methanol (cas number: 67–56-1 Merck-Germany), 2-thiobarbituric acid (cas number: 504–17-6 Merck-Germany), perchloric acid (cas number: 7601–90-3 Sigma-Germany) and 1,1,3,3-tetraethoxypropane (cas number: 122–31-6 Merck-China) were used for MDA and GSH analyses.

Lung tissues were diluted with PBS (pH 7.4) at a ratio of 1:9 and then broken down using a homogeniser. Then, they were centrifuged at 3000 rpm for 20 min and the supernatants were separated and made ready for analysis. MDA levels, one of the important markers of lipid peroxidation, were measured by spectrophotometric determination of the absorbance of the pink colour formed as a result of the reaction using thiobarbituric acid reaction at 532 nm wavelength according to the method of Placer et al. (1966). GSH levels were measured according to the method of Sedlak and Lindsay (1968), which is based on the spectrophotometric determination of the absorbance of the yellow colour formed by the reaction of 2-nitrobenzoic acid at 412 nm. MDA and GSH levels were given as nmol/g. Bcl-2-associated X protein (Bax) (Sunred-China, 202,311), B cell lymphoma 2 (Bcl-2) (Elabscience-USA, JXWE5Z34YC) and caspase-3 (Sunlong-China, SL0152Ra) levels were determined in lung tissue homogenates using commercial Elisa kits.

### Histopathological analyses

The rats were necropsied and the lung tissues were fixed in 10% neutral formalin solution. The tissues were subjected to routine alcohol-xylol follow-up procedures and placed in paraffin blocks. Sections of 4 μ on slides were stained with haematoxylin–eosin and evaluated semiquantitative as absent (0), mild (1), moderate (2) and severe (3) in terms of bronchus-associated lymphoid tissue (BALT) hyperplasia, perivascular cell infiltration and interstitial pneumonia.

### Immunohistochemical analyses

After putting the 4 μm sections on polylysine slides through a series of xylol and alcohol treatments, they were cleaned with PBS and incubated in 3% H_2_O_2_ for 10 min to deactivate endogenous peroxidase. The tissues were subjected to two 5-min treatments of a 500-W antigen retrieval solution in order to expose the antigen within the tissues. Next, they were exposed to IL-1β (Santa Cruz, Catalogue no: sc-52012) and NFκB (Abcam, Catalogue no. ab7971) primary antibodies (dilution 1/200) for an entire night. Secondarily, large volume detection system: anti-polyvalent, HRP (Thermofischer, Catalogue no: TP-125-HL) was applied as recommended by the manufacturer and utilised as a chromogen was AEC (3-Amino-9-Ethylcarbazole). They were examined and covered with aqueous mounting medium after being counterstained with Mayer’s haematoxylin. There were four levels of immunoreactivity assessment: none (0), mild (1), moderate (2) and severe (3).

### Statistical analysis

The data obtained were analysed with SPSS 27.00 software. Independent sample *t* test was used to compare the initial and final body weights of rats, one-way analysis of variance (ANOVA) and Tukey test were used to determine the changes in lung weights and other biochemical parameters. In the evaluation of the data obtained from histopathological and immunohistochemical examinations, the difference between the groups was determined by Kruskal–Wallis, which is a nonparametric test, and Mann–Whitney *U* test was used to determine the group that caused the difference. Values are given as mean + standard deviation and considered significant when *p* < 0.05.

## Results

**Fig. 1:**
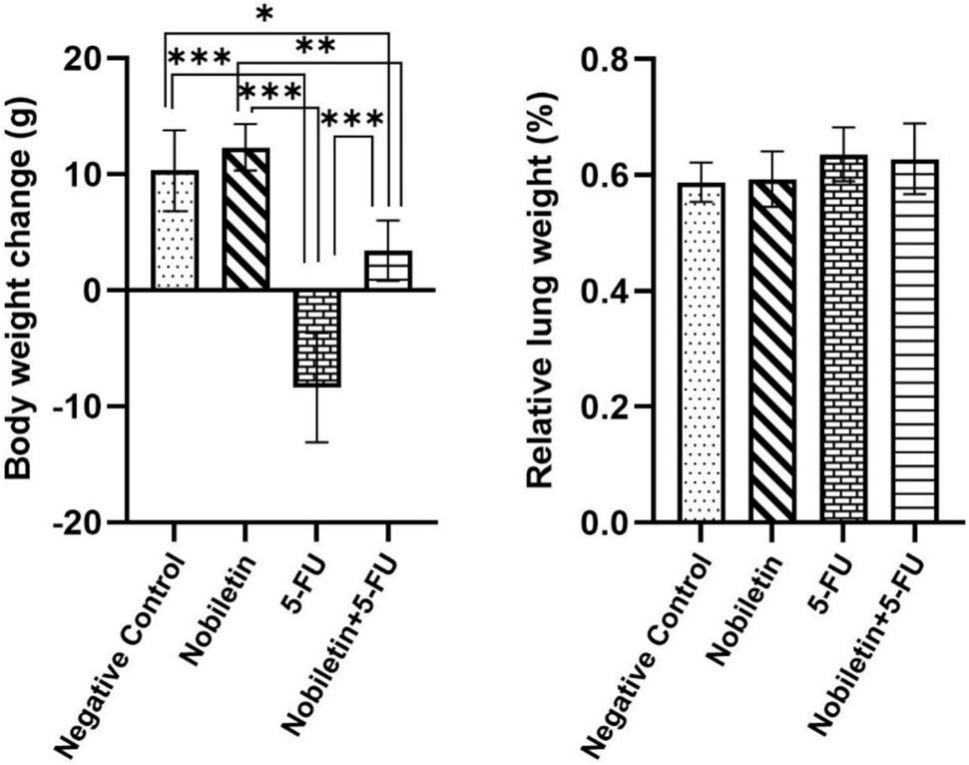
Body weight changes (g) and relative lung weights (%) of the negative control group and experimental groups. *: *p* < 0.05, **: *p* < 0.01, ***: *p* < 0.001

At the beginning of the study, the body weights of the animals were similar and there was no statistical difference between the groups. At the end of the study, changes in body weight were determined by calculating the difference between the measured body weights and the initial weights. The results obtained showed that weight gain was significantly inhibited in the 5-FU group compared to both the negative control group and the Nobiletin group (*p* < 0.001). Inhibition of weight gain in this group indicates that 5-FU causes developmental disorders. Although the weight gain of animals in the Nobiletin + 5-FU group was lower than in the negative control (*p* < 0.05) and Nobiletin groups (*p* < 0.01), it was significantly higher than in the 5-FU group (*p* < 0.001). There was no significant difference between the relative lung weights determined at the end of the study. However, it was noteworthy that lung weights tended to increase in the 5-FU-treated groups (Fig. 1).

**Fig. 2:**
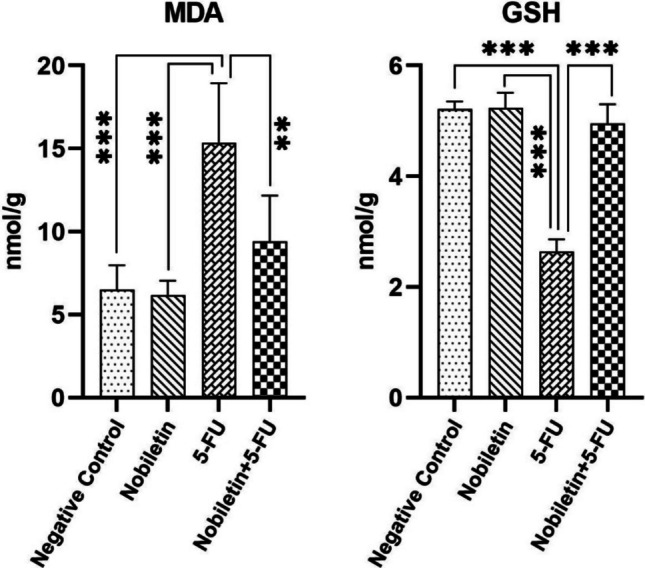
MDA and GSH levels in lung tissue of the negative control group and experimental groups. **p < 0.01, ***: *p* < 0.001

Significantly higher MDA levels in the 5-FU treated group compared to the negative control group (*p* < 0.001) and significantly lower GSH levels (*p* < 0.001) in lung tissue homogenates show that oxidative stress and subsequent oxidative damage increased in the tissue, weakening antioxidant defence (Fig. 2). On the contrary, nobiletin application showed strong antioxidant effects by preventing the increase in MDA levels and decrease in GSH levels (*p* < 0.001).

**Fig. 3:**
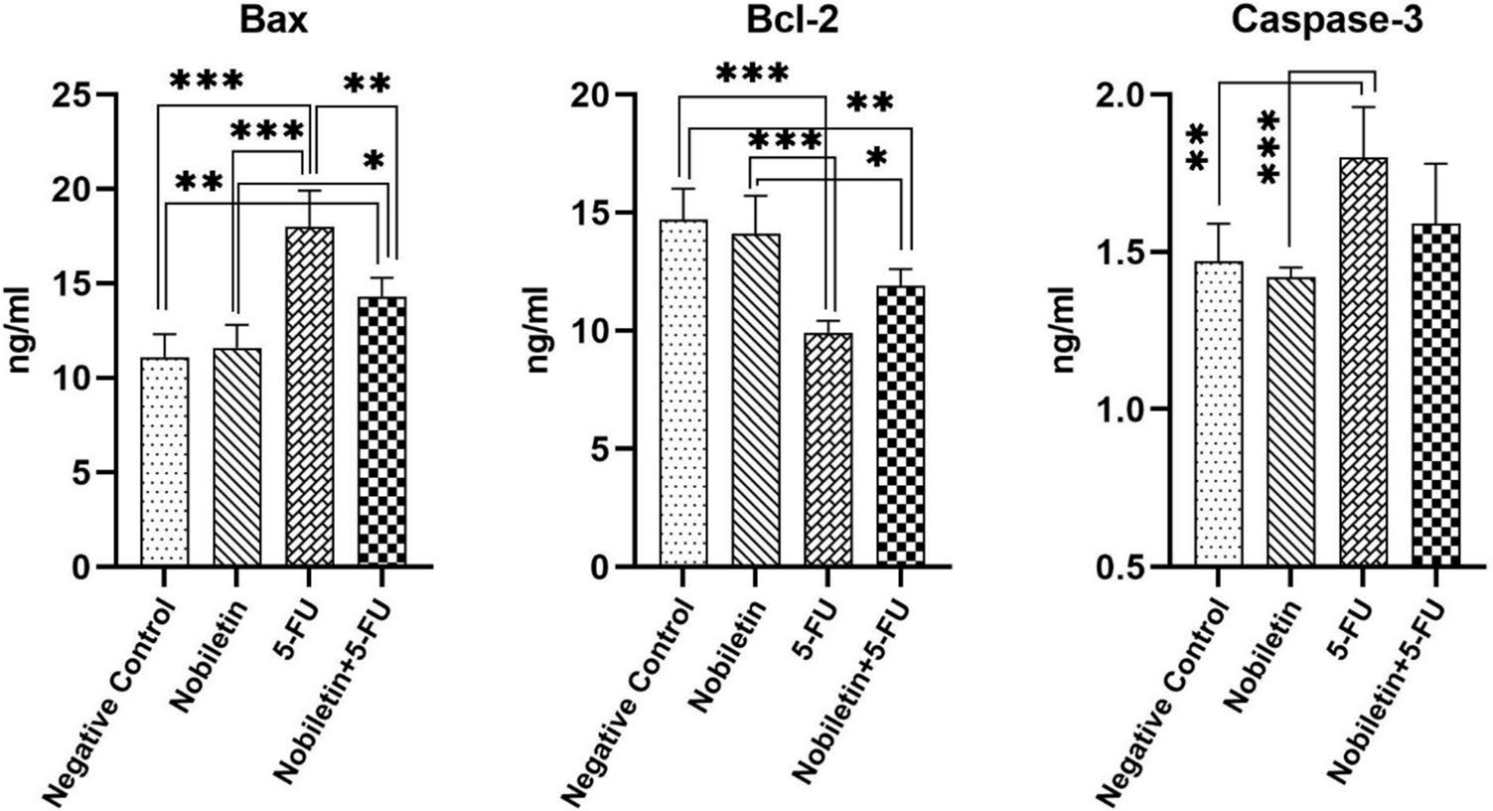
Bax, Bcl-2 and caspase-3 levels in lung tissue of the negative control group and experimental groups. *: *p* < 0.05, **: *p* < 0.01, ***: *p* < 0.001

When the 5-FU group was compared to the negative control group and Nobiletin groups, it was found that the 5-FU group had significantly higher levels of Bax (*p* < 0.001) and caspase-3 (*p* < 0.01, *p* < 0.001, respectively), while Bcl-2 (*p* < 0.001) levels were significantly lower. The Bax level in the Nobiletin + 5-FU group was significantly lower (*p* < 0.01) than in the 5-FU group. Although not statistically different, it was noteworthy that there were some decreases in caspase-3 levels and some increases in Bcl-2 levels in Nobiletin + 5-FU group compared to 5-FU group (Fig. 3).

**Fig. 4:**
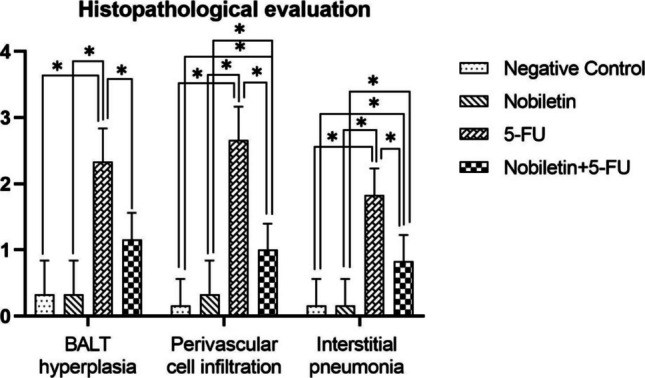
Histopathological evaluation of negative control and experimental groups. *: *p* < 0.05

**Fig. 5:**
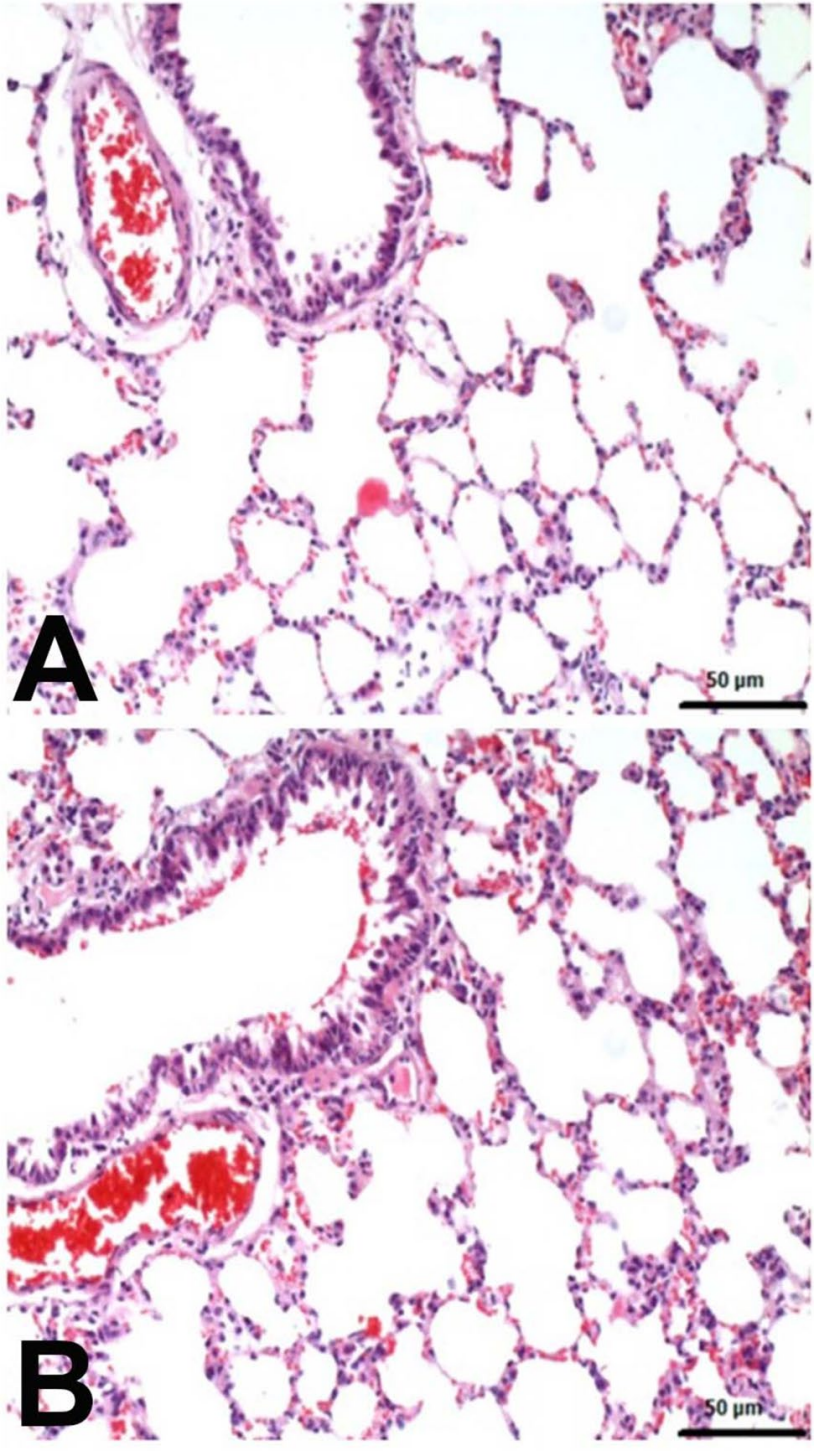
**A** Negative control group. **B** Nobiletin group. Normal histological appearance

**Fig. 6:**
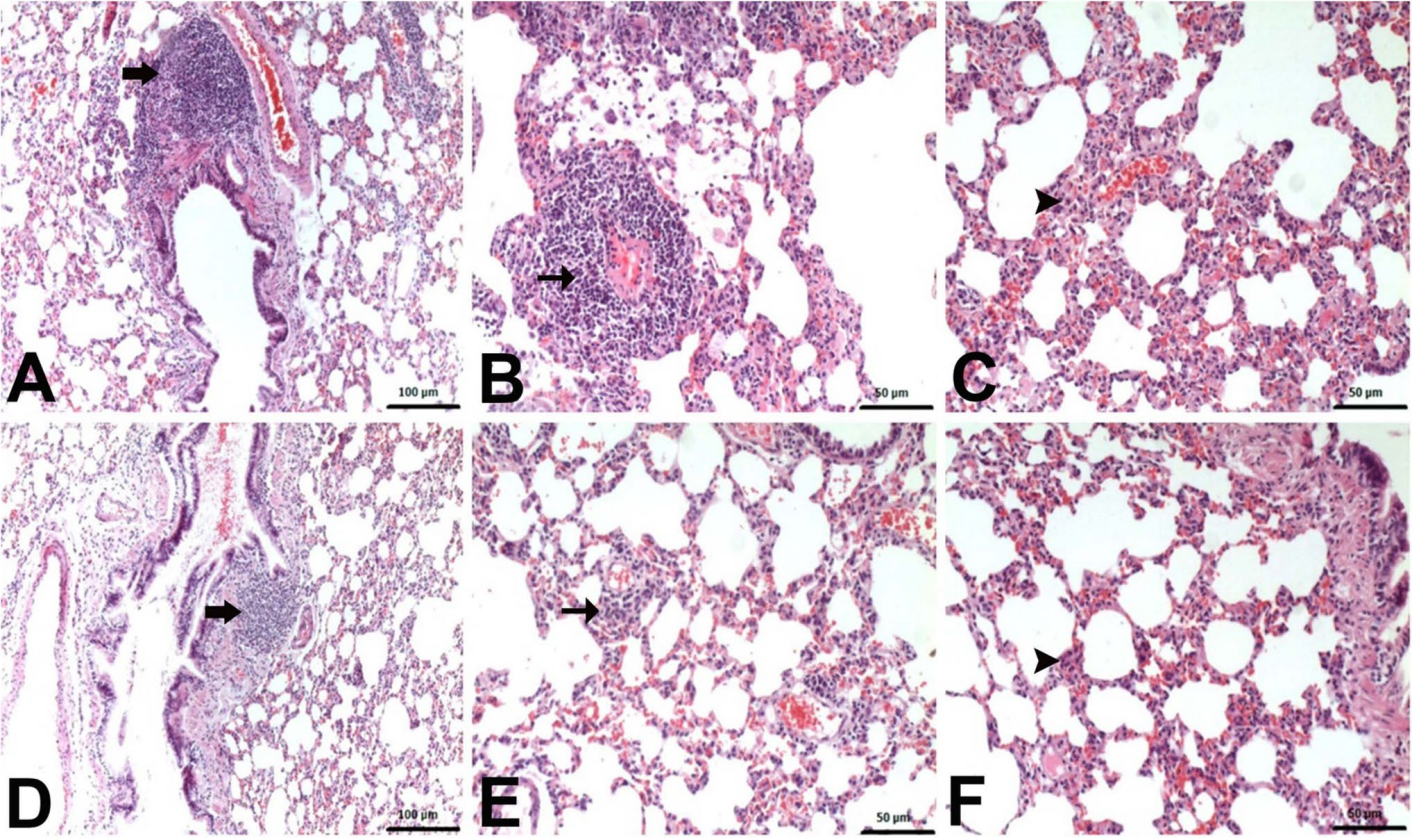
**A**–**C** 5-FU group. Mild BALT hyperplasia (thick arrow), severe perivascular mononuclear cell infiltrations (arrow) and interstitial pneumonia (arrowhead). **D**–**F** Nobiletin + 5-FU group. Normal BALT (thick arrow), moderate perivascular mononuclear cell infiltrations (arrow) and interstitial pneumonia (arrowhead). H-E

Histopathological examinations revealed statistically significant differences between the groups (Fig. 4). Lung samples of the negative control group and Nobiletin group rats had normal histological appearance (Fig. 5). BALT hyperplasia, perivascular cell infiltration and interstitial pneumonia were detected at different levels in 5-FU and Nobiletin + 5-FU groups. Among these histopathological findings, BALT hyperplasia was mild in the 5-FU group, whereas it was not detected at a significant level in the Nobiletin + 5-FU group. Perivascular cell infiltrations and interstitial pneumonia were severe in the 5-FU group and mild in the Nobiletin + 5-FU group (Fig. 6).

**Fig. 7:**
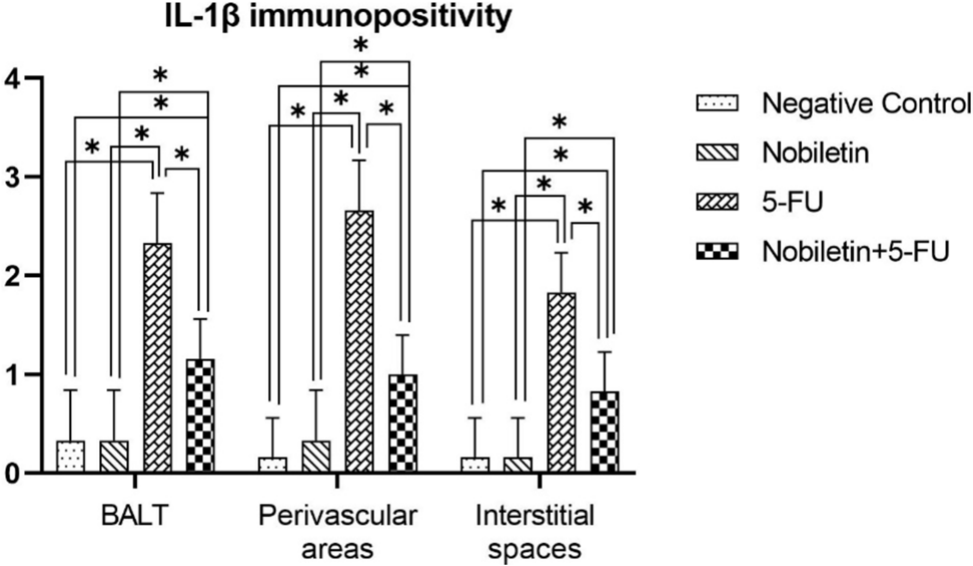
Statistical analysis of IL-1β immunopositivity. *: *p* < 0.05

**Fig. 8:**
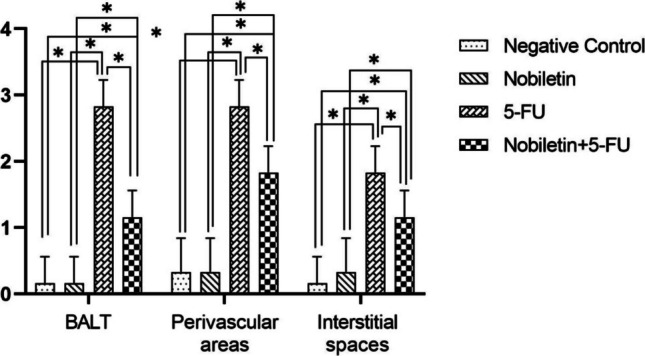
Statistical analysis of NFκB immunopositivity. *: *p* < 0.05

**Fig. 9:**
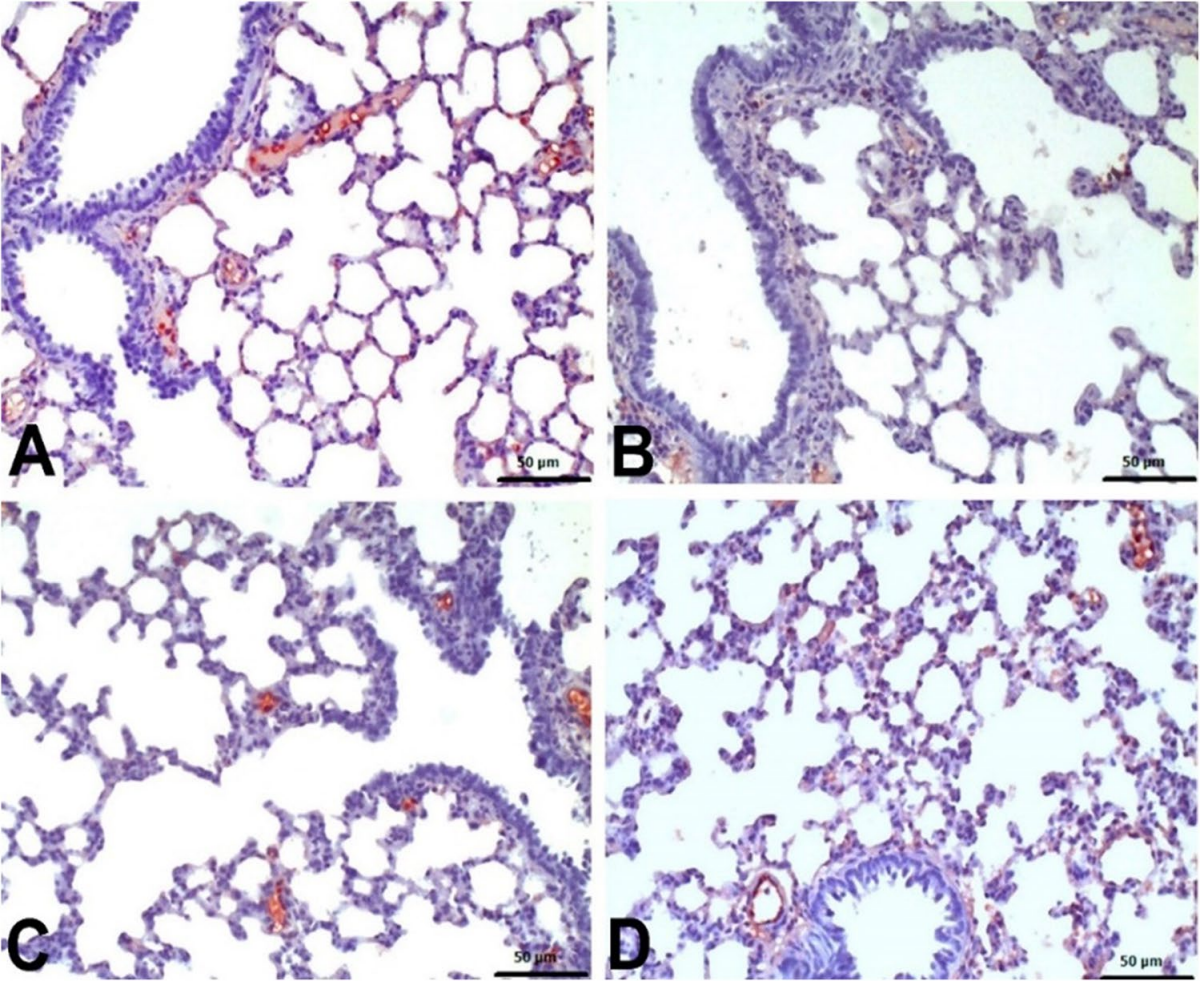
**A** Negative control group IL-1β, **B** Negative control group NFκB, **C** Nobiletin group IL-1β, **D** Nobiletin group NFκB. (IL-1β and NFκB immune negativities.) IHC

**Fig. 10:**
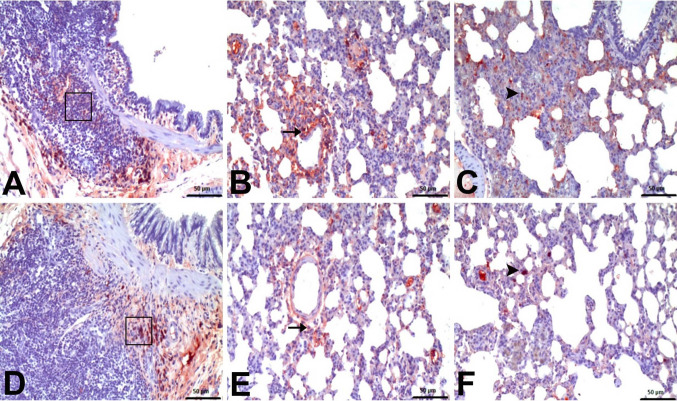
IL-1β immunopositivity. **A** Negative control group, 5-FU group. **A** moderate level (□) in BALT, **B** severe level (arrow) in perivascular areas, **C** moderate level (arrowhead) in interstitial areas. **D**–**E** Nobiletin + 5-FU group: **D** mild in BALT (□), **E** mild in perivascular areas (arrow), **F** mild in interstitial areas (arrowhead), IHC

**Fig. 11:**
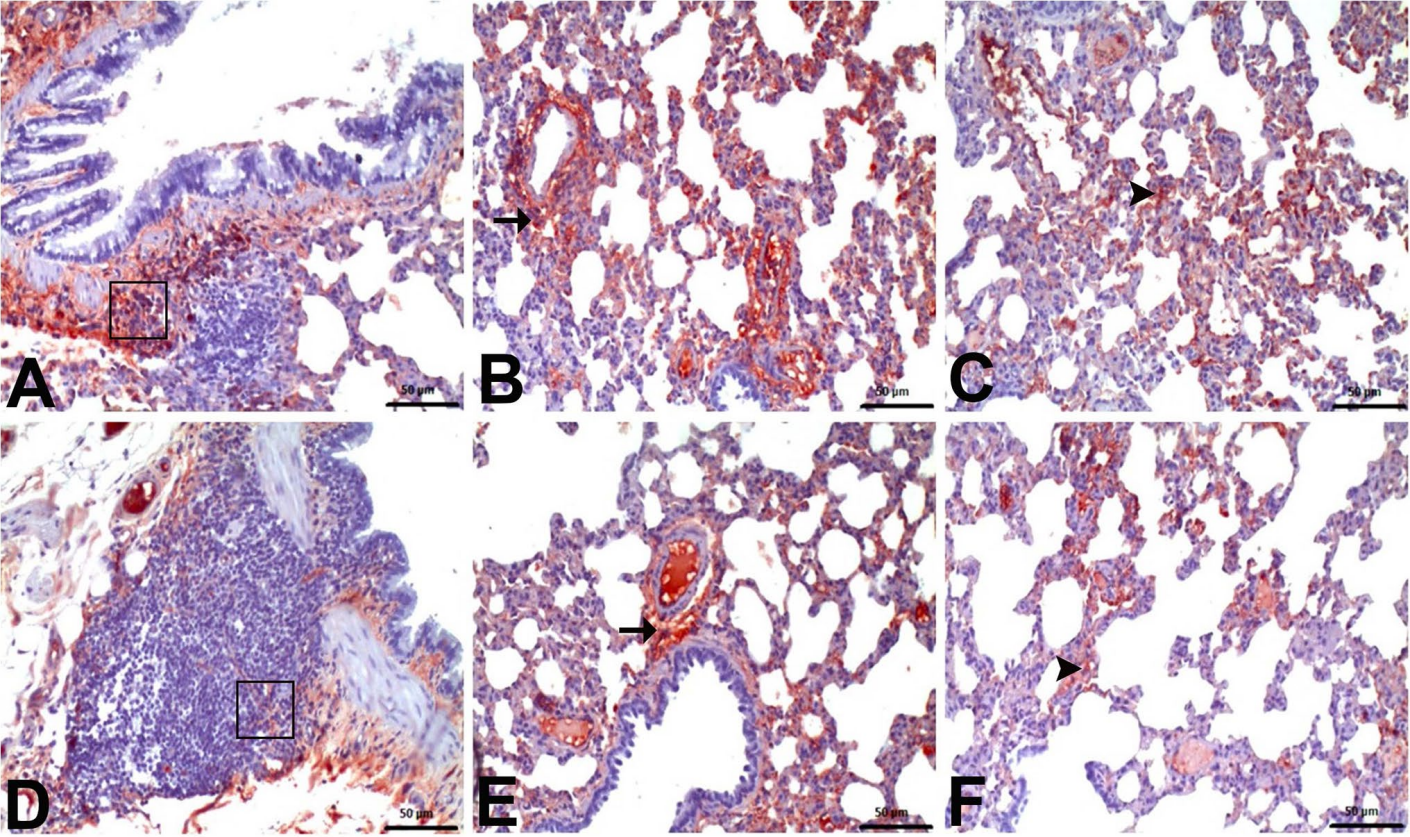
NFκB immunopositivity. **A**–**C** 5-FU group: **A** severe in BALT (□), **B** severe in perivascular areas (arrow), **C** moderate in interstitial areas (arrowhead). **D**–**E** Nobiletin + 5-FU group: **D** mild in BALT (□), **E** moderate in perivascular areas (arrow), **F** mild in interstitial areas (arrowhead), IHC

Immunohistochemical examinations for IL-1β and NFκB revealed statistically significant differences between the groups (Figs. 7 and 8). No significant level of IL-1β and NFκB immunopositivity was detected in the lungs of the negative control group and Nobiletin group rats (Fig. 9). In the other treatment groups, 5-FU group and Nobiletin + 5-FU group, different levels of immunopositivity were detected (Fig. 10). IL-1β immunopositivity showed moderate positivity in BALT and interstitial areas and severe positivity in perivascular areas in the 5-FU group, while mild positivity was observed in these areas in the Nobiletin + 5-FU group (Fig. 10). NFκB immunopositivity in the 5-FU group showed severe positivity in BALT and perivascular areas, while moderate positivity was detected in interstitial areas. In the Nobiletin + 5-FU group, it was determined that NFκB immunopositivity started to attenuate. In this group, NFκB immunopositivity was determined as moderate in perivascular areas and mild in BALT and interstitial areas (Fig. 11).

## Discussion

Generally, the data obtained in this study showed that 5-FU, an important chemotherapeutic agent, causes lung problems such as hyperplasia, cell infiltration and pneumonia in addition to developmental disorders, disrupts the oxidant-antioxidant balance in favour of oxidants, triggers inflammation by increasing NFκB and IL-1β levels, and stimulates apoptosis by increasing Bax and caspase-3 levels and decreasing Bcl-2 levels. On the other hand, it was determined that nobiletin, given as a prophylactic, supports normal development, alleviates the aforementioned lung problems, prevents the disruption of the oxidant-antioxidant balance, reduces inflammation in lung tissue and also exhibits anti-apoptotic effects.

Safarpour et al. (2022a, b, 2023) reported that 5-FU at a dose of 100 mg/kg administered to rats at the beginning of the study prevented weight gain and even significantly decreased body weight at the end of the 14th day. They stated that this decrease in body weight was due to the fact that 5-FU caused growth retardation. Al-Hamdany and Al-Hubaity (2014a) reported that 5-FU at a dose of 20 mg/kg administered to rats for 7 days significantly decreased body weight compared to healthy controls. In another study, it was emphasised that 5-FU administered to rats at doses of 10 mg/kg and 20 mg/kg caused inhibition in the percentage of weight gain even 20 days after administration and this led to growth retardation (El-Sayyad et al. 2009). In this study, when the changes in the body weights of the animals were considered, it was determined that the healthy controls (negative control group and Nobiletin groups) continued their normal development, whereas the development of the 5-FU treated groups (5-FU and Nobiletin + 5-FU) was interrupted. However, the fact that the animals in the Nobiletin + 5-FU group had a positive weight change compared to those in the 5-FU group shows that Nobiletin is effective in preventing the negative effects caused by 5-FU.

5-FU clearance capacity was shown to be lower in females compared to males and not affected by age, while treatment with 5-FU caused stronger haematological toxicity in a female xenograft mouse model compared to a male xenograft mouse model. It was also reported that Korean female patients receiving 5-FU chemotherapy had more frequent side effects such as alopecia and leucopenia compared to male patients and that the risk of increased toxicity to 5-FU treatment was higher in women (Milano et al. 1992; Lim et al. 2019). In another study, gastrointestinal toxicity in males and hematologic toxicity in females were reported during 5-FU treatment and it was emphasised that male and female patients should be evaluated separately (Wettergren et al. 2012). Koga et al. (2007) observed that sex differences were not significant in the effects of nobiletin on liver microsomal metabolism in rats, hamsters and guinea pigs. In this study, female rats were preferred because of the common side effects of 5-FU, it was not considered as a biological factor and sex differences were not addressed. However, this difference is a limitation in terms of both 5-FU and nobiletin that can be considered in future related studies.

Da Silva et al. (2023) administered 15 mg/kg 5-FU to rats for 4 days, 6 mg/kg for the next 4 days and 15 mg/kg on the 14th day of the study and reported that the relative lung weight of 5-FU treated rats was higher than healthy controls. They emphasised that this increase in lung weight was due to the increase in oedematous areas in the lung tissue. In our study, although the lung weights of the 5-FU-treated groups tended to increase, there was no statistical difference between the groups. Possibly an increase in the time after 5-FU injection would have made this change more likely. Al-Hamdany and Al-Hubaity (2014a) reported that 5-FU caused BALT proliferation, mononuclear inflammatory cell infiltration and structural changes in the pulmonary interstitium. They also stated that 5-FU may cause interstitial pneumonia in some studies (Yamane et al. 2013; Kurakawa et al. 2001). In the present study, 5-FU caused mild BALT hyperplasia, severe perivascular mononuclear cell infiltration and interstitial pneumonia. In this respect, our results are consistent with the literature. In the present study, moderate IL-1β immunopositivity was found in BALT and interstitial areas and severe IL-1β immunopositivity was found in perivascular areas. However, severe NF-ĸB immunopositivity was observed in BALT and perivascular areas and moderate NF-ĸB immunopositivity was observed in interstitial areas. In contrast, nobiletin administration normalised BALT appearance and alleviated both mononuclear cell infiltration and interstitial pneumonia formation. In addition, immunohistochemical analyses showed that nobiletin treatment significantly decreased IL-1β immunopositivity in BALT, perivascular and interstitial areas compared to the 5-FU group, while NFκB immunopositivity was significantly attenuated in BALT and interstitial areas and reduced to moderate levels in perivascular areas. Nobiletin is known to have strong antioxidant, anti-inflammatory, antimicrobial, cardioprotective, antidiabetic, neuroprotective effects as well as cancer suppression and even protection of normal cells against toxic agents (Moazamiyanfar et al. 2023; Yang et al. 2020; Wu et al. 2015; Uslu 2021; Lu et al. 2010). Thus, we attribute our results to these previously reported characteristics of nobiletin.

As a result of the literature review, it was observed that the toxicity of 5-FU on lung tissue has not been sufficiently investigated. However, 5-FU may cause severe emphysema, mononuclear cell infiltration, less surfactant release from damaged type-2 alveolar cells, structural disorder and loss of alveolar walls and pulmonary oedema, especially in long-term use. In addition, 5-FU may cause significant lung tissue damage by causing increased NFκB immunopositivity (Vemula et al. 2024). 5-FU may also lead to increase in superoxide anions and decrease in antioxidant enzymes, increase in lipid peroxidation, DNA damage, increase in proinflammatory cytokines and lung toxicity and even apoptosis by stimulation of NFκB signalling pathway (Gelen et al. 2021). Bi et al. (2016) reported that in isoflurane-induced oxidative stress and inflammation, nobiletin showed antioxidant effects by increasing the decreased glutathione peroxidase, GSH and SOD levels and decreasing the increased MDA levels, anti-inflammatory effects by decreasing the increased levels of NFκB, TNF-α, IL-1β and IL-6, and significant apoptotic effects by decreasing Bax levels. Kim et al. (2022) claimed that the anti-carcinogenic effects of Nobiletin against triple-negative breast cancer when administered alone or in combination are due to its regulatory effect on the ROR-IκBα/NFκB pathway and that it has good potential for therapeutic strategy in cancer and inflammation. In another study, nobiletin was reported to inhibit both cancer cell migration and invasion by inhibiting autophagy, G0/G1 cell cycle arrest and NFκB signalling pathway in human pancreatic carcinoma cells (Jiang et al. 2020). Malik et al. (2015) showed that antioxidant levels (GSH, SOD and CAT) decreased and oxidant levels (MDA) increased in cisplatin-induced renal damage, whereas Nobiletin, which was administered protectively, restored the oxidant-antioxidant balance. Vemula et al. (2024) reported that lung tissue GSH and SOD levels decreased and thiobarbituric acid reactive substances levels increased significantly in 5-FU-induced toxicity model, and TNF-α, IL-6, transforming growth factor-β and IL-1β proinflammatory cytokines increased significantly and anti-inflammatory cytokine Interleukin-10 decreased significantly. Similarly, in the present study, we found that MDA levels were significantly increased and GSH levels were significantly decreased in lung tissue due to toxic effects caused by 5-FU. However, we observed that nobiletin given as a preservative before 5-FU administration was highly effective in reversing these levels.

It was reported that Nobiletin showed significant anti-apoptotic effects by decreasing the expression of apoptotic Bax and caspase-3 markers, which were increased, and significantly increasing the expression of anti-apoptotic Bcl-2, which was decreased (Malik et al. 2015). As it is known, increased Bax and caspase-3 levels promote cell apoptosis, while increased Bcl-2 levels inhibit apoptosis. Nobiletin can induce cellular apoptosis in lung cancer by increasing the level of pro-apoptotic protein Bax, decreasing the level of anti-apoptotic protein Bcl-2 and stopping the cell cycle in G2/M phase (Luo et al. 2008; Nagappan et al. 2016). Nobiletin can also prevent cell proliferation by blocking the cell cycle in G1 phase in breast cancer cell lines (MDA-MB-435 and MCF-7) without inducing apoptosis (Morley et al. 2007). However, this is not the case in normal cells. Nobiletin can significantly reduce the production of reactive oxygen species in normal cells and also prevent cellular apoptosis (Yang et al. 2020; Liu et al. 2019). Xu et al. (2020) reported that NOB inhibited the proliferation and migration of renal cell carcinoma by suppressing JAK2/STAT3 and PI3K/Akt pathways and furthermore stimulated their apoptosis. In the present study, 5-FU exhibited strong apoptotic effects in lung tissue by increasing Bax and caspase-3 levels and decreasing Bcl-2 levels, whereas Nobiletin, which was applied as a preservative, showed significant effects in preventing apoptosis, especially by decreasing Bax levels and partially by regulating caspase-3 and Bcl-2 levels. Although nobiletin has anti-apoptotic activity, it promotes apoptosis to prevent the spread of cancer cells and eliminate them.

In a study evaluating the metabolism and oral bioavailability of nobiletin and tangeretin, the major polymethoxylated flavones in citrus fruits, after administration of nobiletin and tangeretin to rats, these compounds and their main metabolites were monitored by high-performance liquid chromatography-electrospray ionisation-mass spectrometry (HPLC–ESI–MS) and eight metabolites of nobiletin and two metabolites of tangeretin were detected. When tangeretin and nobiletin were compared with the same oral doses, it was reported that nobiletin showed approximately tenfold higher absorption compared to tangeretin. In addition, low levels of nobiletin and tangeretin and their metabolites could be detected in rats blood serum even 24 h after treatment (Manthey et al. 2011). However, in clinical drug development, a significant number of potential compounds suffer from solubility and bioavailability difficulties. Nobiletin has been found to have a wide range of bioactivities, including antioxidant, antimicrobial, anticancer and anti-inflammatory, using in vivo experimental models and in vitro studies. However, the low aqueous solubility of nobiletin may cause biopharmaceutical limitations in terms of absorption and bioavailability. Therefore, focus should be placed on improving the formulation of nobiletin so that it can be used effectively in clinical use. Advanced formulations and technologies may provide advantages in revealing its therapeutic effect in the future.

## Conclusion

In this study, it was determined that 5-FU caused severe lung damage by causing oxidative damage, triggering inflammation and inducing cellular apoptosis. However, nobiletin given as a prophylactic agent exhibited anti-inflammatory effects via NFκB pathway and was effective in modulating cellular apoptosis in addition to its antioxidant effect. Because of these beneficial effects, we believe Nobiletin may be a good natural alternative source for reducing the side effects of chemotherapy.

## Data Availability

All source data for this work (or generated in this study) are available upon reasonable request.
