# Correction: Nobiletin reduces 5-FU-induced lung injury with antioxidative, anti-inflammatory and anti-apoptotic activities

**DOI:** 10.1007/s00210-025-04878-2

**Published:** 2026-03-06

**Authors:** Gözde Atila Uslu, Hamit Uslu, Taha Abdulkadir Çoban, Mustafa Özkaraca, Ali Sefa Mendil, Serpil Aygörmez

**Affiliations:** 1 Department of Physiology, Faculty of Medicine, Erzincan Binali Yıldırım University, Erzincan, Turkey; 2 Department of Biochemistry, Faculty of Medicine, Erzincan Binali Yıldırım University, Erzincan, Turkey; 3 Department of Pathology, Faculty of Veterinary Medicine, Cumhuriyet University, Sivas, Turkey; 4 Department of Pathology, Faculty of Veterinary Medicine, Erciyes University, Kayseri, Turkey; 5 Department of Biochemistry, Faculty of Veterinary Medicine, Kafkas University, Kars, Turkey

**Correction: Naunyn-Schmiedeberg's Archives of Pharmacology (2025) 398:8543-8554**

10.1007/s00210-024-03773-6

In the originally published version of this article, inaccuracies were identified in Figure 9. Specifically, the images in Figures 9C and 9D were incorrectly presented due to the misplacement of the representative image for Figure 9D. This error has now been corrected, and Figure 9D has been revised accordingly.

Additionally, errors were found in the annotation of Figure 9. Figure 9B was mistakenly labeled as the Nobiletin group; however, it actually represents the negative control group (NFκB). Conversely, Figure 9C was incorrectly labeled as the negative control group, whereas it should be identified as the Nobiletin group (IL-1β). The legend of Figure 9 has been corrected to accurately reflect these revisions.

These corrections do not affect the results or conclusions of the study. The corrected figure and legend are provided below.

**Figure:**
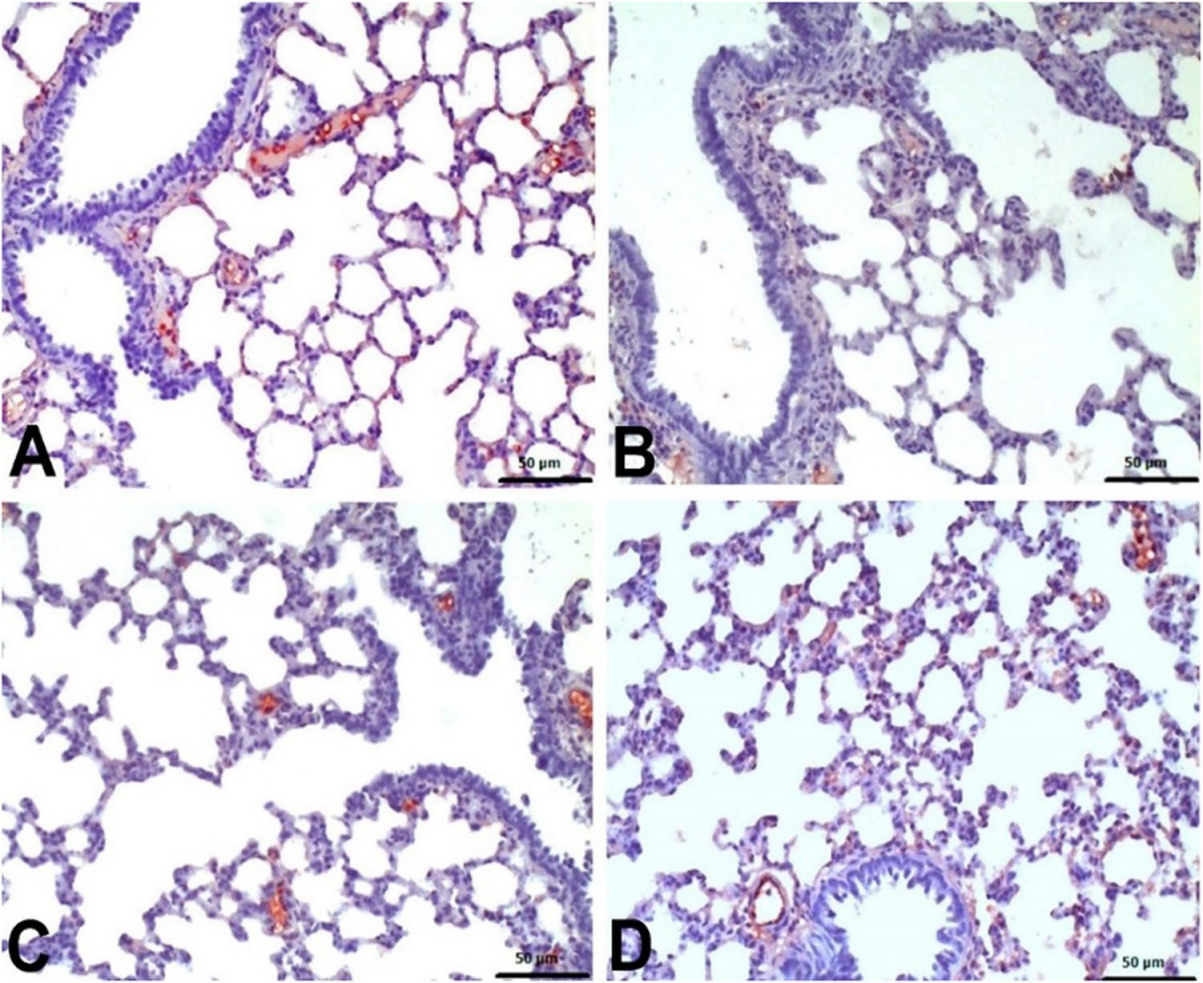
**Fig. 9 A **Negative control group IL-1β, **B** Negative control group NFκB, **C** Nobiletin group IL-1β, **D** Nobiletin group NFκB. (IL-1β and NFκB immune negativities.) IHC

